# Supplementary material for: Identifying latent genetic interactions in genome-wide association studies using multiple traits
Source: Genome Med. 2024 Apr 25;16:62. doi: 10.1186/s13073-024-01329-0 (PMC11044415; doi:10.1186/s13073-024-01329-0)
Supplement: Supplementary file 1 — Additional file 1. Supplementary methods. Theoretical results for latent interaction testing. Figure S1. General strategy to detect latent genetic interactions when there are two unobserved environments. Figure S2. Revealing latent interactive effects using multiple traits. Figure S3. False positive rate of the LIT implementations under the null hypothesis of no interaction. Figure S4. Q-Q plot of the LIT implementations under the null hypothesis of no interaction. Figure S5. False positive rate of the LIT implementations when applied to 5 SNPs. Figure S6. The empirical power of the principal components for the squared residual and cross product matrix at various baseline correlations. Figure S7. Comparing uLIT, wLIT, and aLIT when the direction of the effect size for the interaction term is opposite of the environmental variable. Figure S8. Comparing Levene’s test to aLIT, Marginal (SQ/CP), and Marginal (SQ) when the direction of the effect size for the interaction term is the same as the environmental variable. Figure S9. Comparing Levene’s test to aLIT, Marginal (SQ/CP), and Marginal (SQ) when the direction of the effect size for the interaction term is opposite of the environmental variable. Figure S10. Quantile-Quantile plot of the uLIT, wLIT, and aLIT p-values from the UK Biobank. Figure S11. Quantile-Quantile plot of the Marginal (SQ) p-values from the UK Biobank using the traits BFP, BMI, HC and WC. Figure S12. The genomic inflation factor as a function of minor allele frequency in the UK Biobank analysis. Figure S13. The double KS test procedure using the set of independent SNPs. Figure S14. Comparison of the significance results using the marginal testing procedure and aLIT. Figure S15. Comparison of aLIT p-values after adjusting for additive genetic effects and dominance/scaling effects. Figure S16. Simulation results using a Gaussian kernel in LIT when the direction of the effect size for the interaction term is the same as the environmental variable. Fi [file 13073_2024_1329_MOESM1_ESM.pdf]

Supplementary materials: Identifying latent genetic interactions in genome-wide association studies using multiple traits

Andrew J. Bass[1,*], Shijia Bian[2], Aliza P. Wingo[3], Thomas S. Wingo[1,4], David J. Cutler[1] and Michael P. Epstein[1,*]

[1] *Department of Human Genetics, Emory University, Atlanta, GA 30322, USA*
[2] *Department of Biostatistics and Bioinformatics, Emory University, Atlanta, GA 30322, USA*
[3] *Department of Psychiatry, Emory University, Atlanta, GA 30322, USA*
[4] *Department of Neurology, Emory University, Atlanta, GA 30322, USA*
[*] *Corresponding authors:* `ajbass@emory.edu`, `mpepste@emory.edu`

Contents

1 Supplementary methods **2**
1.1 Testing for latent genetic interactions . . . 2
1.2 Latent interactions induce differential variance and covariance patterns . . . 2
1.3 Distribution of the cross products . . . 3
1.4 Regression model for the cross products and squared residuals . . . 4

2 Supplementary figures **7**

3 Supplementary tables **25**

1 Supplementary methods

1.1 Testing for latent genetic interactions

To review the regression model from the Results section, suppose Y_{jk} depends on a biallelic locus with genotype X_j, an unobserved (or latent) environmental variable M_j, and a latent genotype-by-environment (GxE) interaction X_jM_j for $j = 1, 2, \ldots, n$ unrelated individuals with $k = 1, 2, \ldots r$ measurable traits. The regression model is expressed as

$$Y_{jk} = \beta_k X_j + \phi_k M_j + \gamma_k X_j M_j + \epsilon_{jk}, \tag{S1}$$

The left side of the equation are the trait values which are observable random variables. The right side contains four components: the observable genotype X_j with effect size β_k; an unobservable variable M_j with effect size ϕ_k; an unobservable interaction X_jM_j with effect size γ_k; and an unobservable random error ϵ_{jk} with mean zero and variance σ_k^2. Without loss of generality, we assume that M_j is mean zero with unit variance. Our inference goal is it to test whether $\gamma_k = 0$ for $k = 1, 2, \ldots, r$ without having to observe the latent environmental variable M_j.

The following sections are outlined as follows. We first show that a latent genetic interaction induces trait variance and covariance patterns under the above model assumptions. We then review the distributional theory behind the individual-level trait central cross moments. Using these results, we briefly show how latent interactive effects can be detected within a regression model framework.

1.2 Latent interactions induce differential variance and covariance patterns

We show in the main text that a latent interaction can be detected based on calculating the individual-specific trait variances (ITV) and covariances (ITC). To construct these quantities, let $e_{jk} = Y_{jk} - \beta_k X_j$ denote the trait residuals after removing the additive genetic effect. For simplicity, assume the effect sizes are known. For the jth individual, given the genotype X_j, the $r \times r$ individual-specific trait covariance matrix is

$$\mathbf{\Sigma}_j \mid X_j = \begin{bmatrix} \mathrm{E}\left[e_{j1}^2 \,\middle|\, X_j\right] & \mathrm{E}[e_{j1}e_{j2} \mid X_j] & \cdots & \mathrm{E}[e_{j1}e_{jr} \mid X_j] \\ \mathrm{E}[e_{j2}e_{j1} \mid X_j] & \mathrm{E}\left[e_{j2}^2 \,\middle|\, X_j\right] & \cdots & \mathrm{E}[e_{j2}e_{jr} \mid X_j] \\ \vdots & \vdots & \ddots & \vdots \\ \mathrm{E}[e_{jr}e_{j1} \mid X_j] & \mathrm{E}[e_{jr}e_{j2} \mid X_j] & \cdots & \mathrm{E}\left[e_{jr}^2 \,\middle|\, X_j\right] \end{bmatrix},$$

where the ITV are the r diagonal elements and ITC are the $s = \binom{r}{2}$ off-diagonal elements.

The presence of a latent interaction shared by multiple traits induces differential ITV (vQTL) and ITC (covQTL) patterns as a function of genotype. More specifically, given our model assumptions, the

ITC between the kth and k'th trait is

$$
\begin{aligned}
\mathrm{Cov}\big[Y_{jk}, Y_{jk'} \bigm| X_j\big] &= \mathrm{E}\big[e_{jk}e_{jk'} \bigm| X_j\big] \\
&= \mathrm{E}\big[(\phi_k M_j + \gamma_k X_j M_j + \epsilon_{jk})(\phi_{k'} M_j + \gamma_{k'} X_j M_j + \epsilon_{jk'}) \bigm| X_j\big] \\
&= \mathrm{E}\big[\phi_k\phi_{k'} M_j^2 + (\phi_{k'}\gamma_k + \phi_k\gamma_{k'})X_j M_j^2 + \gamma_{k'}\gamma_k X_j^2 M_j^2 \bigm| X_j\big] \\
&+ \mathrm{E}\big[\phi_k M_j \epsilon_{jk'} + \gamma_k X_j M_j \epsilon_{jk'} + \phi_{k'} M_j \epsilon_{jk} + \gamma_{k'} X_j M_j \epsilon_{jk} + \epsilon_{jk}\epsilon_{jk'} \bigm| X_j\big] \\
&= \mathrm{E}\big[\phi_k\phi_{k'} M_j^2 + (\phi_{k'}\gamma_k + \phi_k\gamma_{k'})X_j M_j^2 + \gamma_{k'}\gamma_k X_j^2 M_j^2 \bigm| X_j\big] \\
&= \big(\phi_k\phi_{k'} + (\phi_{k'}\gamma_k + \phi_k\gamma_{k'})X_j + \gamma_{k'}\gamma_k X_j^2\big)\,\mathrm{E}\big[M_j^2 \bigm| X_j\big] \\
&= \tilde{a}_{kk'} + \tilde{b}_{kk'} X_j + \tilde{c}_{kk'} X_j^2,
\end{aligned}
\tag{S2}
$$

where $\tilde{a}_{kk'} = \phi_k\phi_{k'}$, $\tilde{b}_{kk'} = \phi_k\gamma_{k'} + \phi_{k'}\gamma_k$, and $\tilde{c}_{kk'} = \gamma_k\gamma_{k'}$. Note that the fourth line follows from our assumption that the random errors of each trait are independent of each other, the genotype, and the environmental variable, and so $\mathrm{E}\big[M_j\epsilon_{jk'} \bigm| X_j\big] = \mathrm{E}[M_j\epsilon_{jk} \mid X_j] = \mathrm{E}\big[\epsilon_{jk}\epsilon_{jk'} \bigm| X_j\big] = 0$. The fifth line follows from the assumption that the environmental variable M_j is mean zero with unit variance and independent of the genotype, and so $\mathrm{E}[M_j \mid X_j] = \mathrm{E}[M_j] = 0$ implying that $\mathrm{E}\Big[M_j^2 \Bigm| X_j\Big] = \mathrm{Var}[M_j \mid X_j] + \mathrm{E}[M_j \mid X_j]^2 = \mathrm{Var}[M_j \mid X_j] = \mathrm{Var}[M_j] = 1$. Following similar steps as above, the ITV is

$$
\begin{aligned}
\mathrm{Var}[Y_{jk} \mid X_j] &= \mathrm{E}\big[e_{jk}^2 \bigm| X_j\big] \\
&= a_k + b_k X_j + c_k X_j^2,
\end{aligned}
\tag{S3}
$$

where $a_k = \phi_k^2 + \sigma_k^2$, $b_k = 2\phi_k\gamma_k$, and $c_k = \gamma_k^2$. Thus, we have shown that a latent GxE interaction will create differential trait variance and covariance patterns that depend on genotype. In particular, a latent GxE interaction in trait k ($\gamma_k \neq 0$) will induce a variance pattern that depends on genotype (Equation S3), and also induce a covariance pattern between traits k and k' when there is a shared interaction ($\gamma_{k'} \neq 0$) or a shared interacting variable ($\phi_{k'} \neq 0$; Equation S2).

Even though we limit our discussion to a single latent environmental effect and genotype for simplicity, our results hold more generally under the polygenic trait model. Furthermore, while we consider a simple interaction effect, it is straightforward to show that other complex latent signals involving the genotype induce differential variance and covariance patterns. Although, the exact functional form may be more complicated than above.

1.3 Distribution of the cross products

Following the above discussion, we describe the distribution for the cross product of two random variables that follow a Normal distribution. We then use this result to describe the sampling variability of the cross product and squared residual terms within a regression model framework in the next section.

To simplify notation, let $Y_1 \equiv Y_{j1}$ and $Y_2 \equiv Y_{j2}$ denote the first two traits of the jth individual. Without loss of generality, suppose these traits are normally distributed with mean zero, unit variance, and correlation coefficient ρ. The cross product term is denoted by $Z = Y_1 Y_2$.

The relationship between traits can be expressed as

$$Y_2 = \rho Y_1 + \sqrt{1-\rho^2} U, \tag{S4}$$

where $U \sim \mathrm{N}(0,1)$. The cross product term is then

$$\begin{aligned} Z &= Y_1(\rho Y_1 + \sqrt{1-\rho^2} U) \\ &= \rho Y_1^2 + \sqrt{1-\rho^2} Y_1 U, \end{aligned} \tag{S5}$$

where $Y_1^2 \sim \chi_1^2$ and $Y_1 U \sim \mathrm{B}_0$ where B_0 is the modified Bessel distribution of the second kind of order zero. For perfectly correlated variables, Z is distributed as a Chi-squared distribution with one degree of freedom. Alternatively, for uncorrelated variables, Z follows a modified Bessel distribution of the second kind of order zero. See ref. [1,2] for the distribution of the product of two normal random variables.

The first two moments are

$$\begin{aligned} \mathrm{E}[Z] &= \rho \\ \mathrm{Var}[Z] &= 1 + \rho^2, \end{aligned} \tag{S6}$$

and, more generally, for mean centered traits with variances (σ_1^2, σ_2^2), the first two moments are

$$\begin{aligned} \mathrm{E}[Z] &= \sigma_1 \sigma_2 \rho \\ \mathrm{Var}[Z] &= \sigma_1^2 \sigma_2^2 (1 + \rho^2). \end{aligned} \tag{S7}$$

We use this result in the next section to describe the heteroskedasticity in a regression model that treats the cross products or squared residuals as outcome variables.

1.4 Regression model for the cross products and squared residuals

Using the central moments result, we first describe the regression model for the cross product terms. Let $P = \{(1,2),(1,3),\ldots,(2,3),(2,4),\ldots,(r-1,r)\}$ denote the set of cross product pairs such that $|P| = s$. The first and second element of the qth cross product is P_{q1} and P_{q2}, respectively, and the cross product between traits is $Z_{jq}^{\mathrm{CP}} = e_{j,P_{q1}} e_{j,P_{q2}}$. The regression model is

$$\begin{aligned} Z_{jq}^{\mathrm{CP}} \mid X_j &= \mathrm{E}\big[Z_{jq}^{\mathrm{CP}} \mid X_j\big] + \epsilon_{jq} \\ Z_{jq}^{\mathrm{CP}} \mid X_j &= \tilde{a}_q + \tilde{b}_q X_j + \tilde{c}_q X_j^2 + \epsilon_{jq}, \end{aligned} \tag{S8}$$

where $\mathrm{E}\Big[Z_{jq}^{\mathrm{CP}} \,\Big|\, X_j\Big] = \mathrm{Cov}[e_{j,P_{q1}}, e_{j,P_{q2}} \mid X_j]$ is expressed in Equation S2. The results in Section 1.3 can be used to describe the random error in the model: The error term ϵ_{jq} is independent for $j =$

$1, 2, \ldots, n$ observations, but in general, is not normally distributed or identically distributed. Under the null hypothesis of no interactive effects, the errors are identically distributed.

We note that the above regression model differs from typical regression models in two ways. First, the random error does not follow a Normal distribution, although for typical large GWAS sample sizes, this should not impact inference. Second, under the alternative hypothesis where interactions exists, heteroskedasticity arises in the model. To see why, using the results from the previous section, the variance of the error term can be expressed as

$$\mathrm{Var}[\epsilon_{jq} \mid X_j] = \sigma^2_{j,Y_{P_{q1}}|X_j} \sigma^2_{j,Y_{P_{q2}}|X_j} + \mathrm{E}\big[Z^{\mathrm{CP}}_{jq} \mid X_j\big]^2 \tag{S9}$$

where $\sigma^2_{Y_{j,P_{q1}}|X_j} = (\phi_{P_{q1}} + \gamma_{P_{q1}} X_j)^2 + \sigma^2_{P_{q1}}$ and $\sigma^2_{Y_{j,P_{q2}}|X_j} = (\phi_{P_{q2}} + \gamma_{P_{q2}} X_j)^2 + \sigma^2_{P_{q2}}$. Under the null hypothesis, if the heteroskedasticity is uncorrelated with the explanatory variables then there is type I error rate control. Therefore, controlling for sources of variation such as population structure and nearby SNPs with strong additive effects is important to avoid an inflated type I error rate. Finally, in addition to these sources of variation, an incorrect trait scaling will likely induce heteroskedasticity and also impact type I error rate control.

We briefly state the regression model using the ITV. For the ITV, we are modeling the change in variance of trait k as a function of X_j:

$$\begin{aligned} Z^{\mathrm{SQ}}_{jk} \mid X_j &= \mathrm{E}\Big[Z^{\mathrm{SQ}}_{jk} \;\Big|\; X_j\Big] + \epsilon'_{jk} \\ Z^{\mathrm{SQ}}_{jk} \mid X_j &= a_k + b_k X_j + c_k X_j^2 + \epsilon'_{jk}, \end{aligned} \tag{S10}$$

where $\mathrm{Var}\Big[\epsilon'_{jk} \;\Big|\; X_j\Big] = 2\sigma^4_{Y_{jk}|X_j}$. The ITVs are a special case of the ITCs when $\rho = 1$.

Thus far, we assumed that the effect sizes of the additive genetic term is known to simplify the theory. However, in practice, we use the residuals so the above theory does not exactly hold: while the studentized residuals are unbiased estimates, they follow a t-distribution and so the squared residuals follow an F-distribution (similar adjustments with the cross products). This nuance did not impact any inferences in our simulation study.

There are a few important details with the above regression model approach. First, a test for differential ITV patterns is related to the Breusch-Pagan test [3]. In addition, a regression model on the correlation scale has been discussed elsewhere (see, e.g., [4]) and, more recently, is related to one studied by Lea et al. (2019) [5]. Second, the quadratic relationship between the cross products (or squared residuals) and genotypes only holds for simple interactions, and the underlying (and unknown) functional form is expected to be more complicated. Regardless, for GWAS data where interactions are difficult to detect, c_q (or c_k) is likely much smaller than b_q (or b_k) and so it is reasonable to assume that the linear term will dominate the signal compared to higher order terms.

References

1. Nadarajah S, Pogány TK. On the distribution of the product of correlated normal random variables. Comptes Rendus Mathematique. 2016;354(2):201–204. doi:https://doi.org/10.1016/j.crma.2015.10.019.
2. Cui G, Yu X, Iommelli S, Kong L. Exact distribution for the product of two correlated Gaussian random variables. IEEE Signal Processing Letters. 2016;23(11):1662–1666. doi:10.1109/LSP.2016.2614539.
3. Trevor S Breusch and Adrian R Pagan. A simple test for heteroscedasticity and random coefficient variation. Econometrica. 1979;47(5):1287–1294.
4. Bartlett RF. Linear modelling of Pearson's product moment correlation coefficient: An application of Fisher's z-transformation. Journal of the Royal Statistical Society Series D (The Statistician). 1993;42(1):45–53.
5. Lea A, Subramaniam M, Ko A, Lehtimäki T, Raitoharju E, Kähönen M, et al. Genetic and environmental perturbations lead to regulatory decoherence. eLife. 2019;8:e40538. doi:10.7554/eLife.40538.

2 Supplementary figures

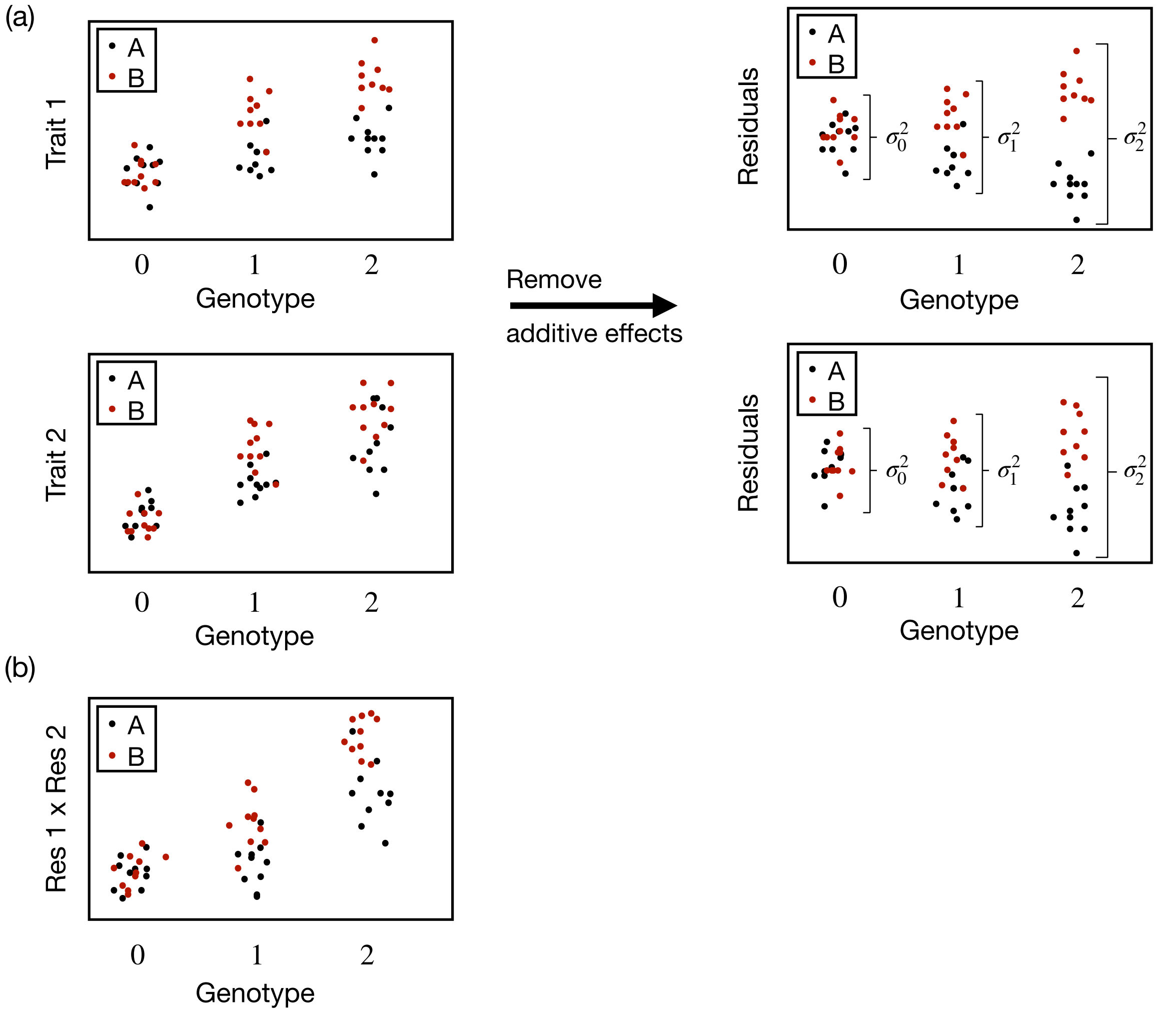

Fig. S1: General strategy to detect latent genetic interactions when there are two unobserved environments denoted by 'A' and 'B.' (a) The additive genetic effect is removed and any heteroskedasticity correlated with genotype implies a latent genetic interaction. (b) When there are two traits measured, the pairwise products between the residuals (cross products) can be used to test for latent genetic effects.

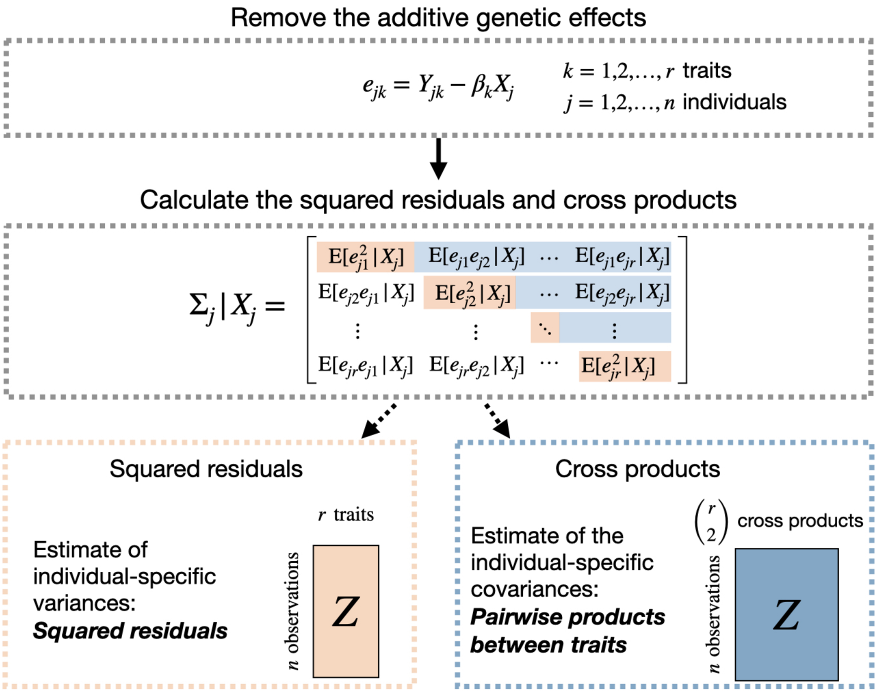

Fig. S2: Revealing latent interactive effects using multiple traits. The first step is to remove the additive genetic signal to ensure that the covariance between traits is not caused by the main (additive) effects of the SNP. The individual-specific covariance matrix can then be estimated by calculating the corresponding squared residuals (estimate of the diagonal elements) and the cross products (estimate of the off-diagonal elements). These quantities can be used to infer latent interactive effects.

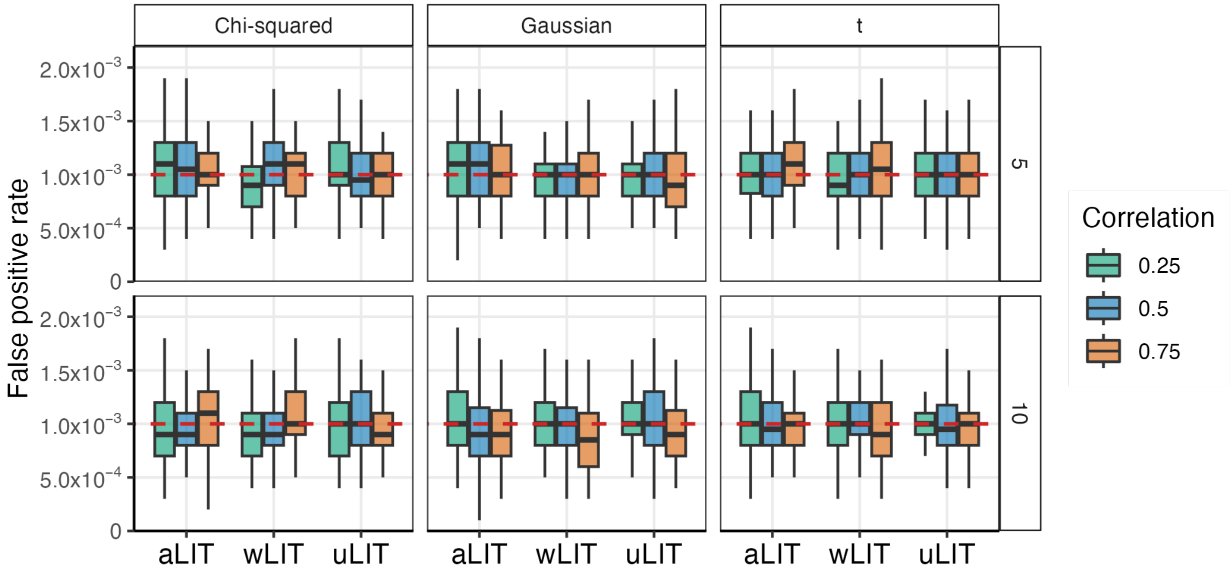

Fig. S3: False positive rate of the LIT implementations under the null hypothesis of no interaction. Our simulation study varied the number of traits (rows), baseline trait correlation (0.25 (green), 0.50 (blue), and 0.75 (orange)), and error distribution (columns). For each configuration, there are 50 replicates at a sample size of $300{,}000$. The empirical false positive rate at a type I error rate of 1×10^{-3} (red dashed line).

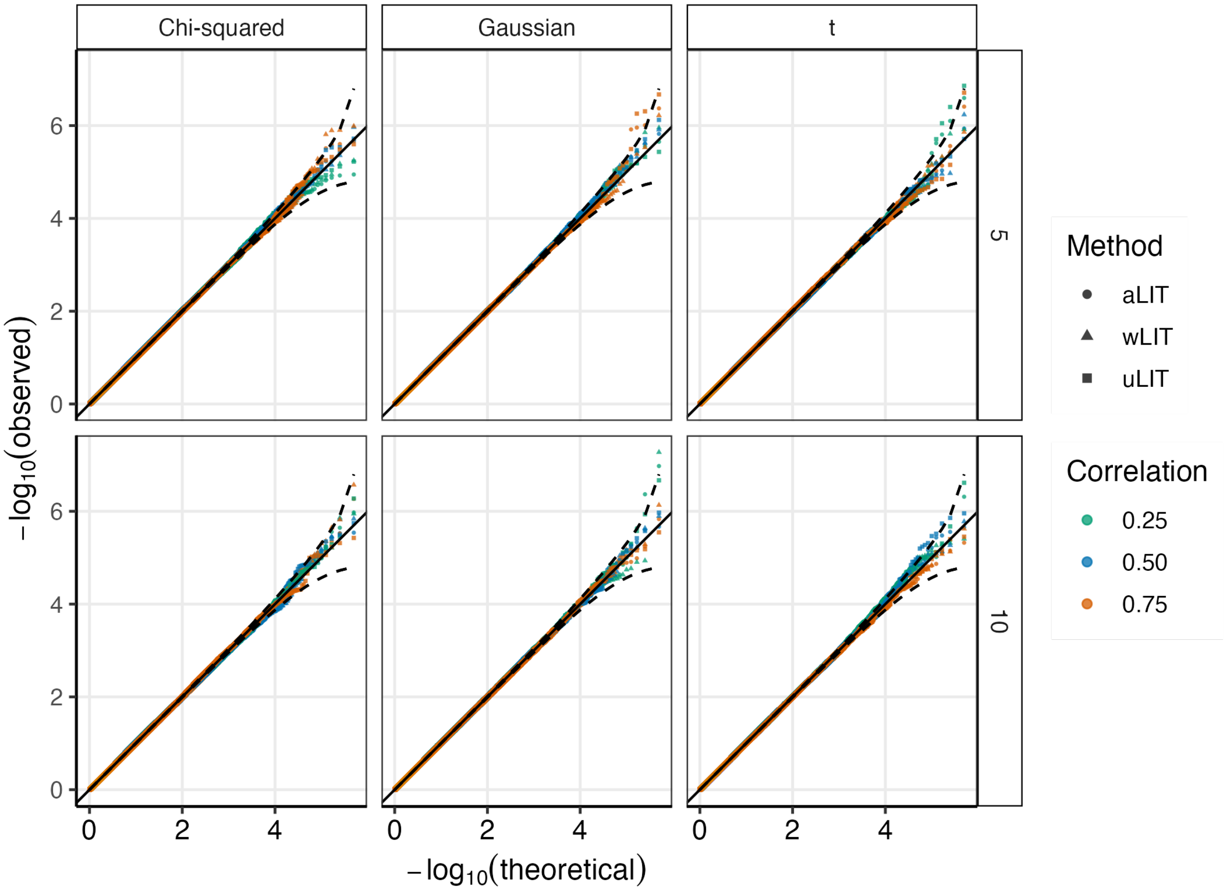

Fig. S4: Q-Q plot of the LIT implementations under the null hypothesis of no interaction. Similar to Figure S3, our simulation study varied the number of traits (rows), baseline trait correlation (0.25 (green), 0.50 (blue), and 0.75 (orange)), and error distribution (columns). At each configuration, we simulated 50 datasets of $10{,}000$ SNPs and then combined the p-values for a total of $500{,}000$ p-values per configuration.

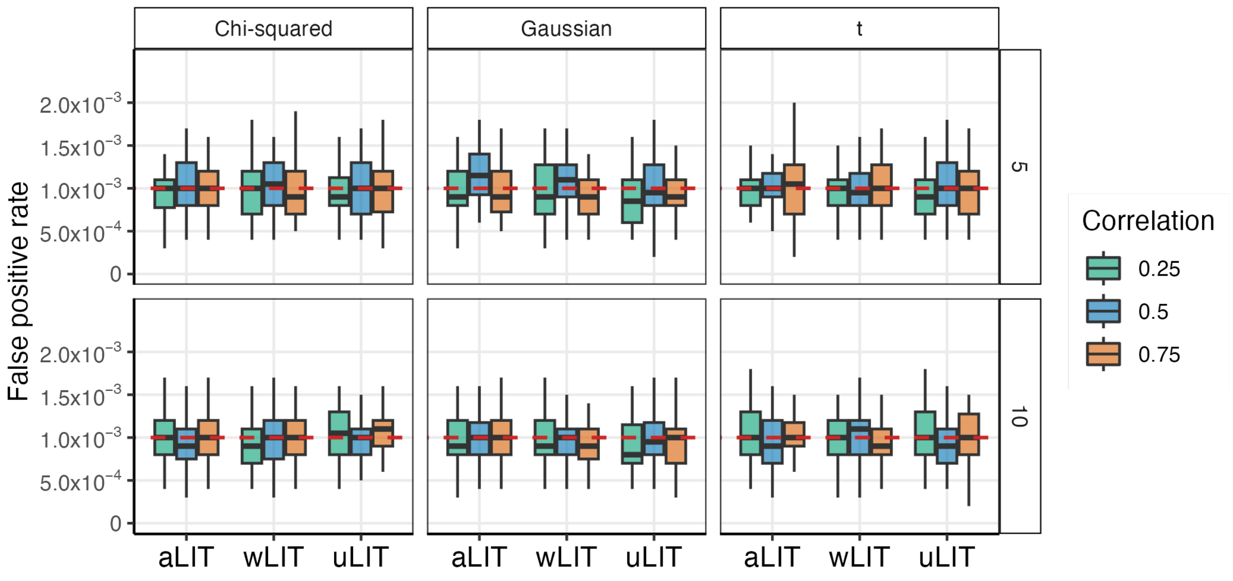

Fig. S5: False positive rate of the LIT implementations when applied to 5 SNPs.

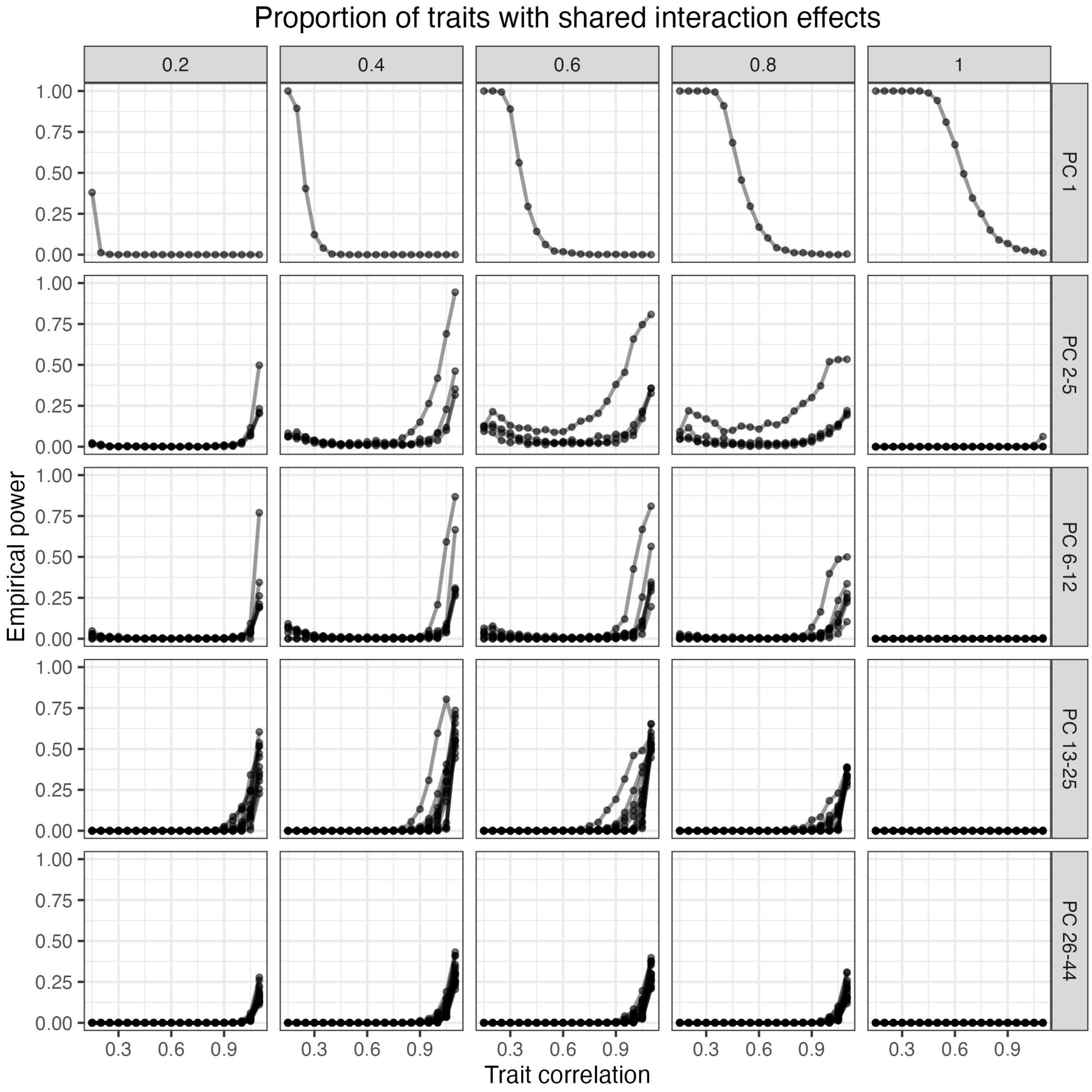

Fig. S6: The empirical power of the principal components (rows) for the squared residual and cross product matrix at various baseline correlations (x-axis). In total, there was 10 traits simulated and the proportion of traits with shared interaction effects (columns) was varied. Each point represents the average power across 500 simulations at a significance threshold of 5×10^{-8}.

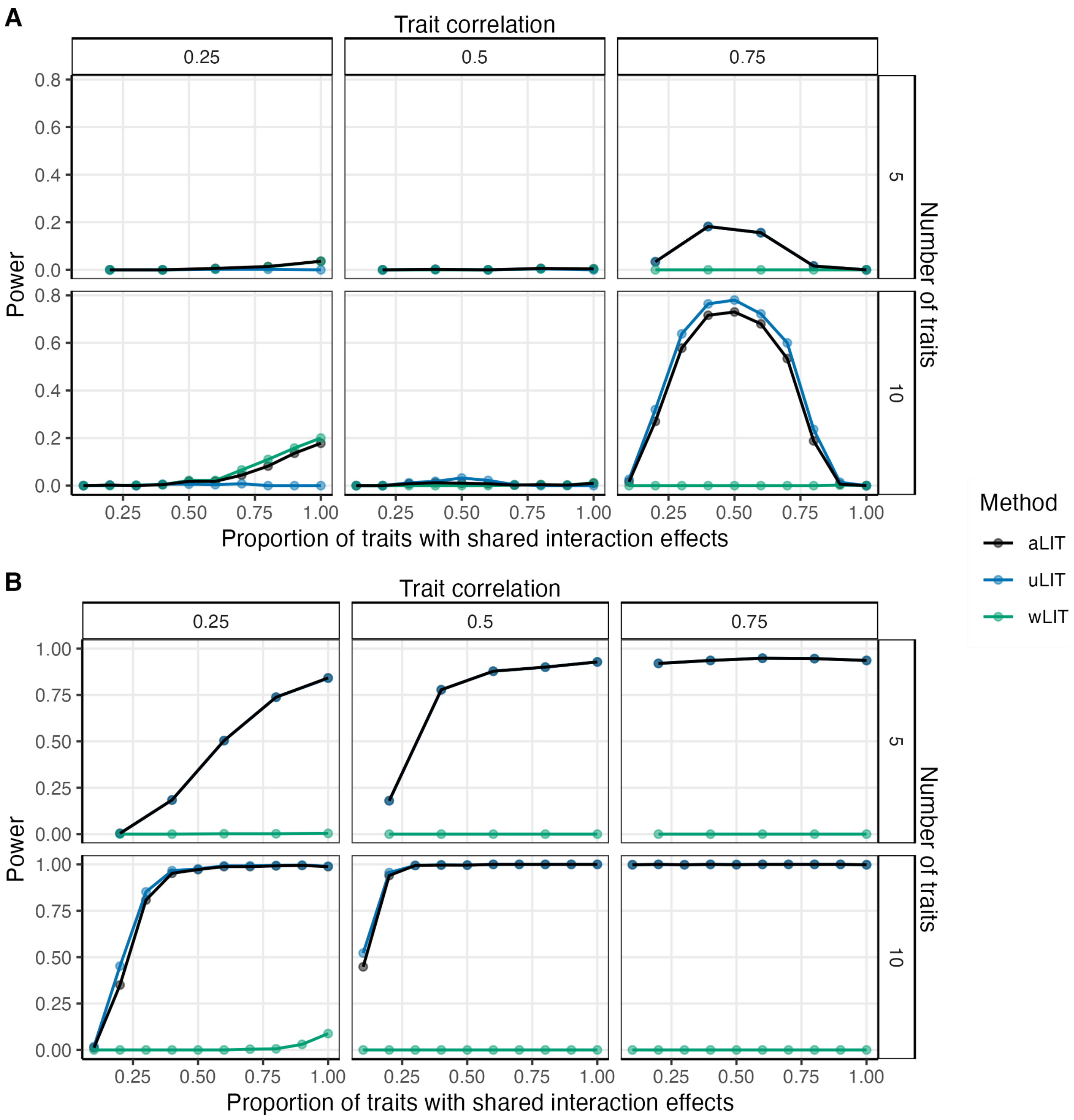

Fig. S7: A similar simulation setting to Figure 2 with the direction of the effect size for the interaction term is opposite of the interacting environmental variable under (A) positive pleiotropy and (B) a mixture of positive and negative pleiotropy.

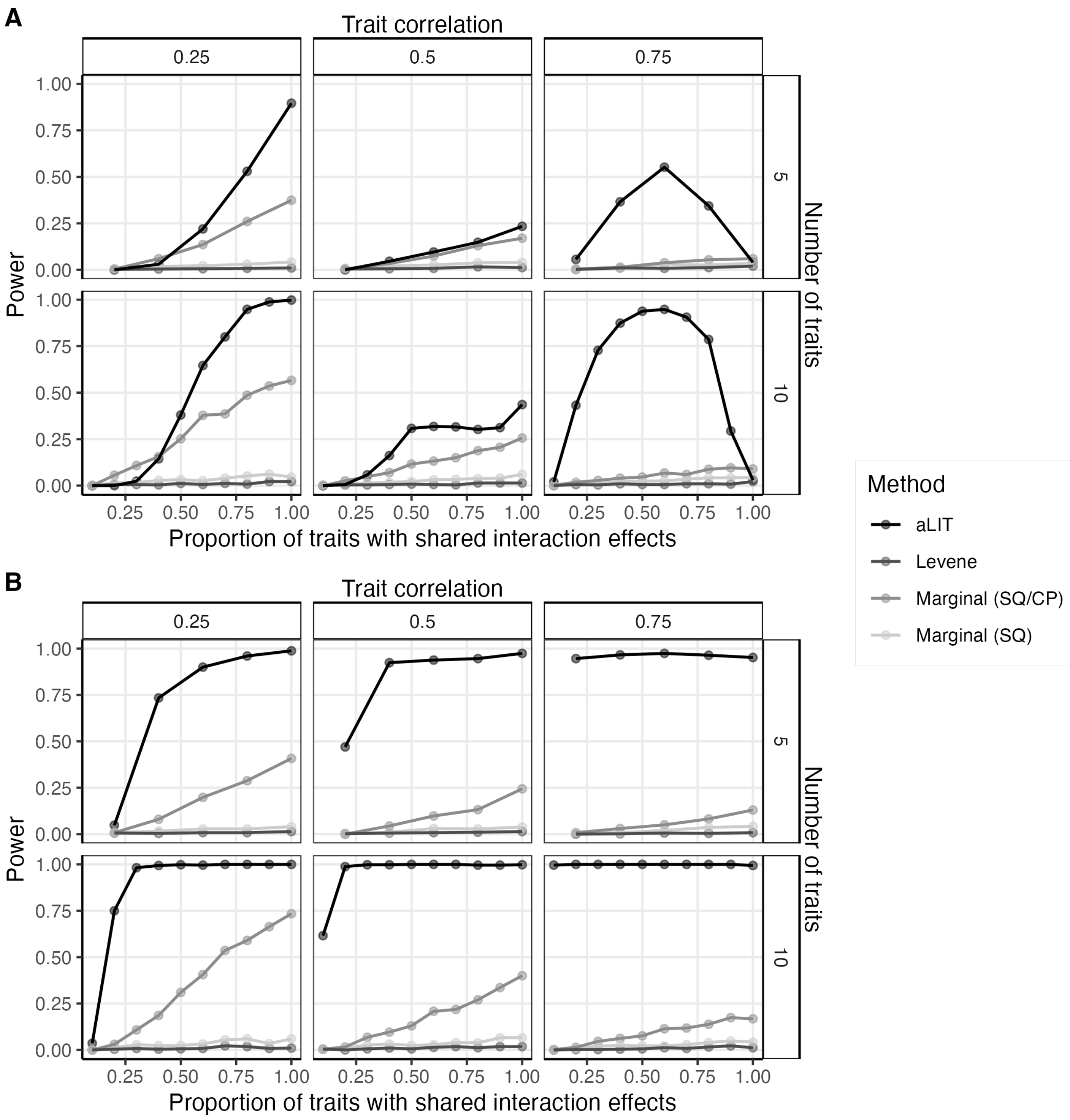

Fig. S8: Comparing Levene's test to aLIT, Marginal (SQ/CP), and Marginal (SQ) using a similar simulation setting to Figure 3.

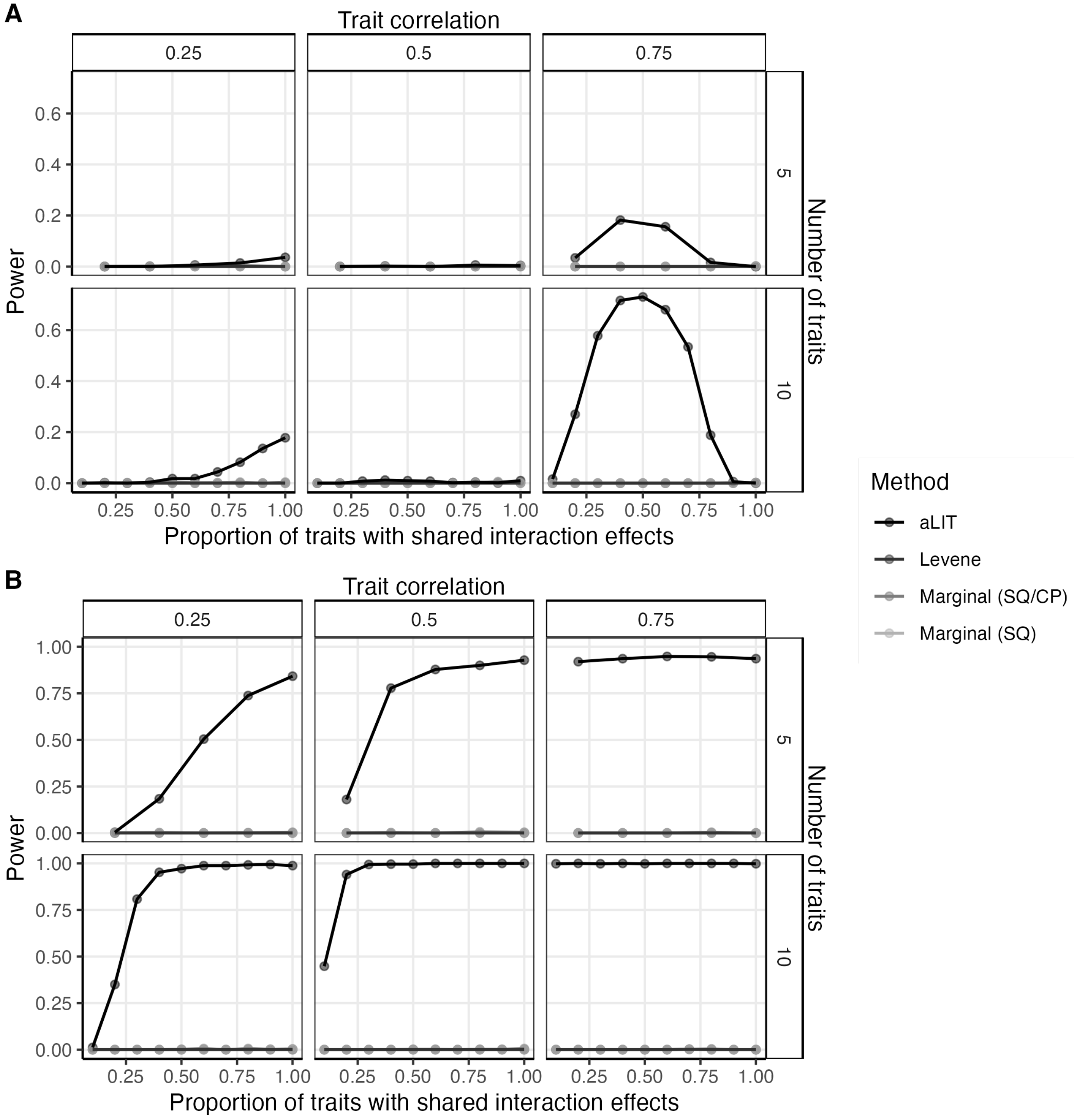

Fig. S9: Comparing Levene's test to aLIT, Marginal (SQ/CP), and Marginal (SQ) using a similar simulation setting to Figure S7.

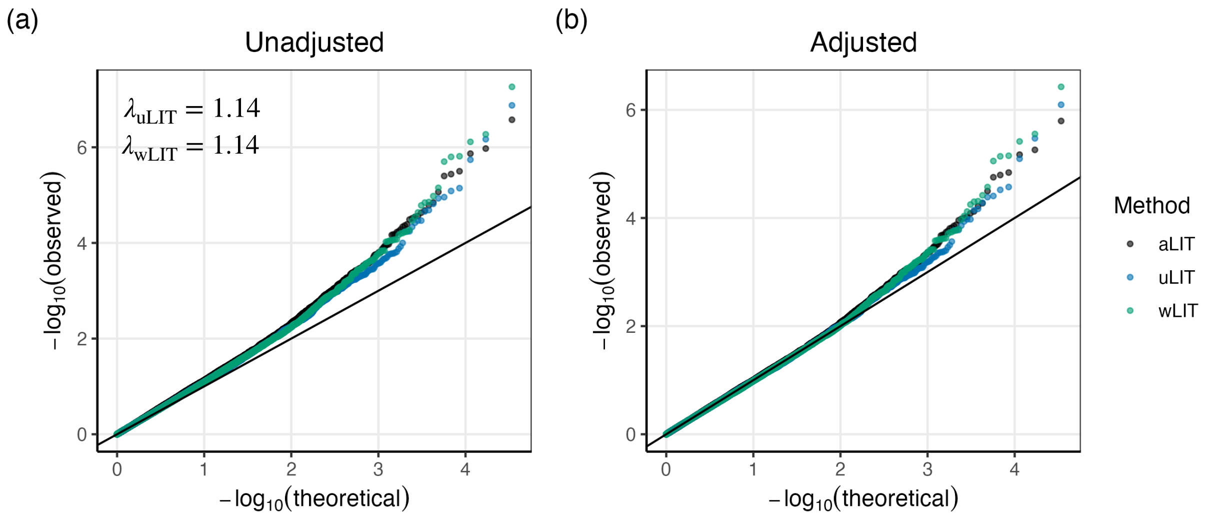

Fig. S10: Quantile-Quantile plot of the uLIT, wLIT, and aLIT p-values from the UK Biobank. (a) The unadjusted p-values and (b) adjusted p-values using the genomic inflation factor. The figure removes significant p-values and those in linkage disequilibrium.

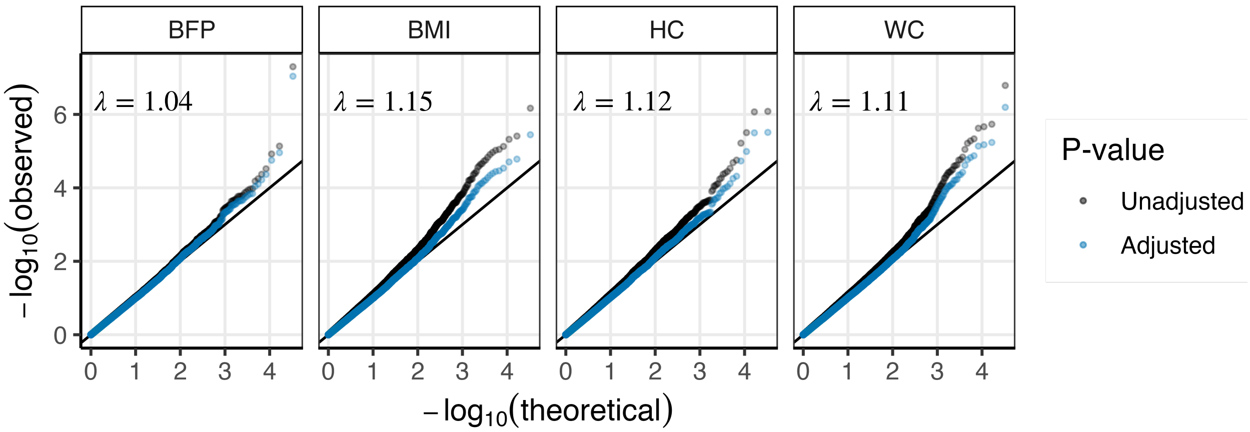

Fig. S11: Quantile-Quantile plot of the Marginal (SQ) p-values from the UK Biobank using the traits BFP, BMI, HC and WC. The unadjusted (blue) and adjusted (black) p-values are shown. We removed the significant p-values and those in linkage disequilibrium.

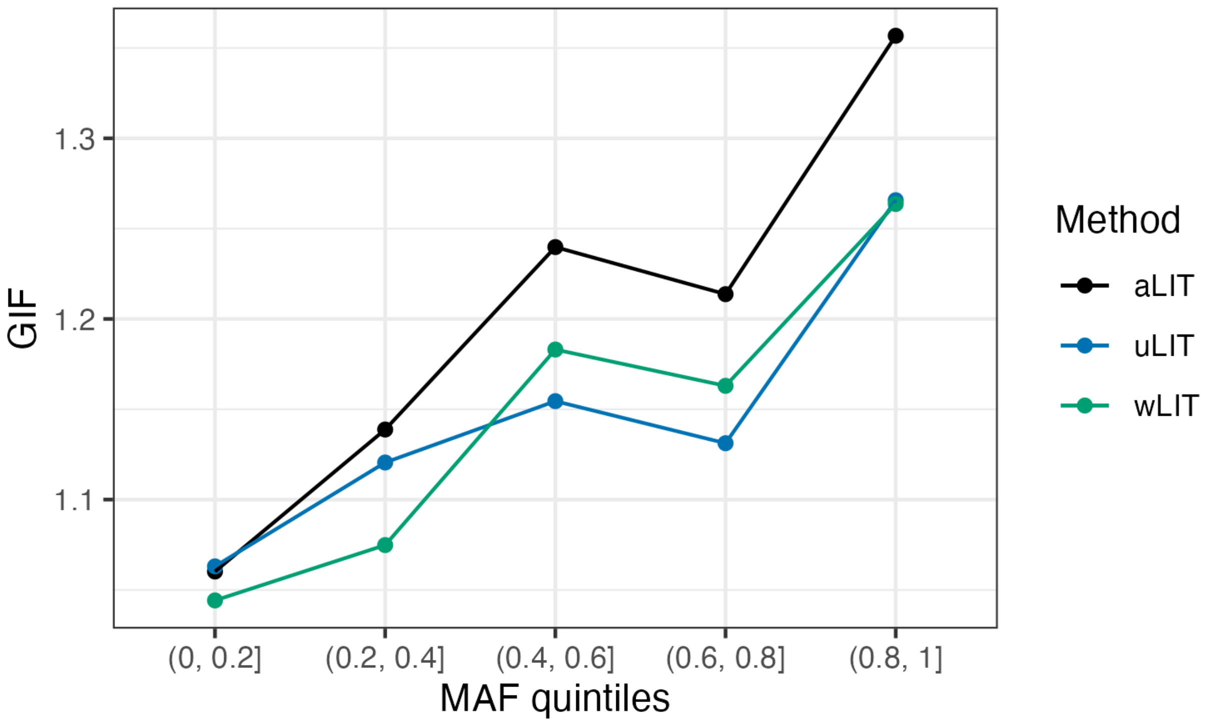

Fig. S12: The observed SNP minor allele frequency (MAF) distribution in the UK Biobank was split into 5 equal parts (quintiles) where the genomic inflation factor (GIF) was calculated for uLIT, wLIT, and aLIT.

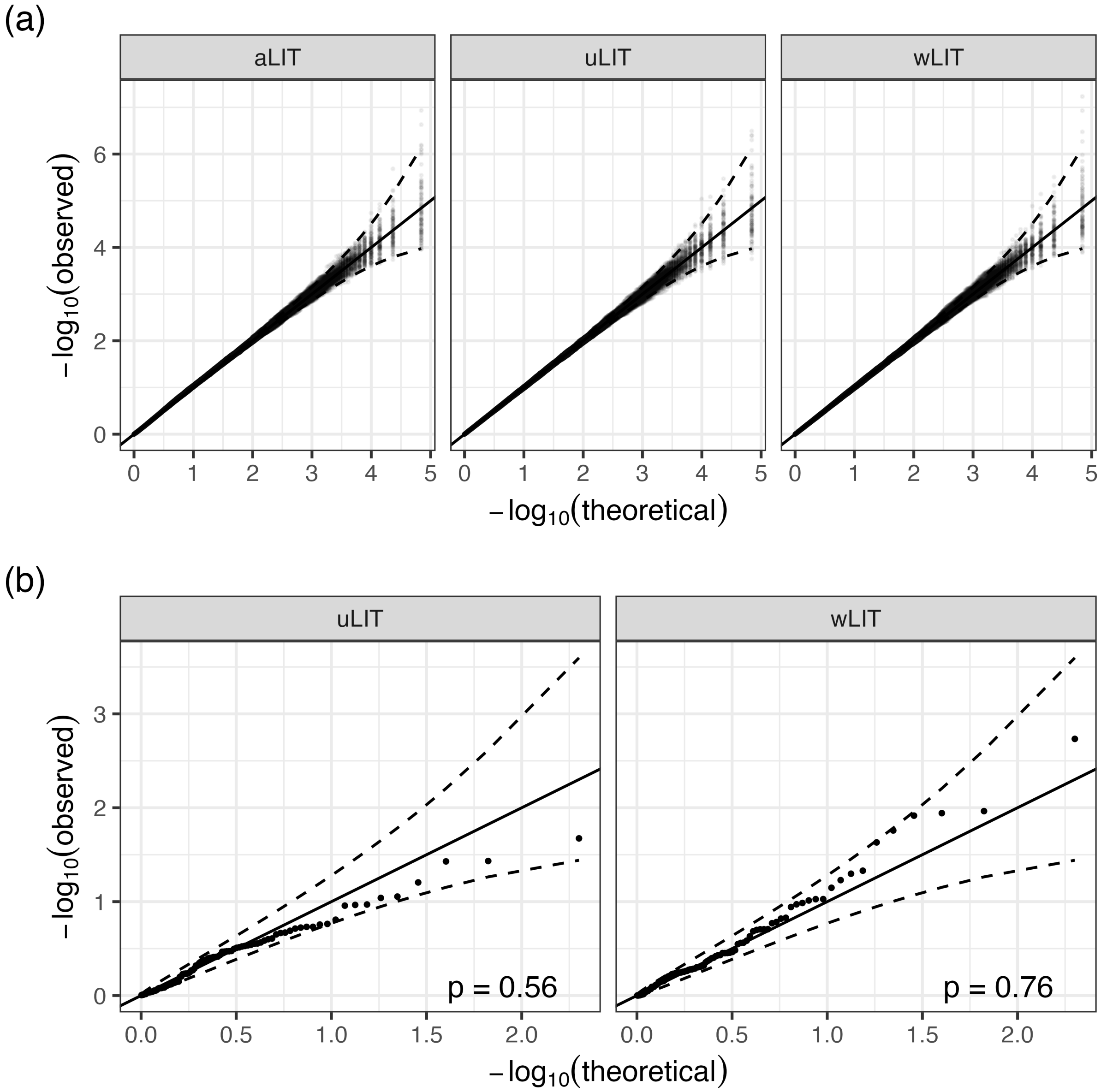

Fig. S13: The double KS test procedure using the set of independent SNPs in Figure S10. (a) Quantile-Quantile plot of aLIT, uLIT, and wLIT p-values from 100 permutations of the phenotype under the null hypothesis of no latent genetic interactions. (b) Quantile-Quantile plot of the 100 KS test p-values for uLIT and wLIT. The double KS test p-value is shown in the bottom right corner. The dashed line indicates the 95% confidence band.

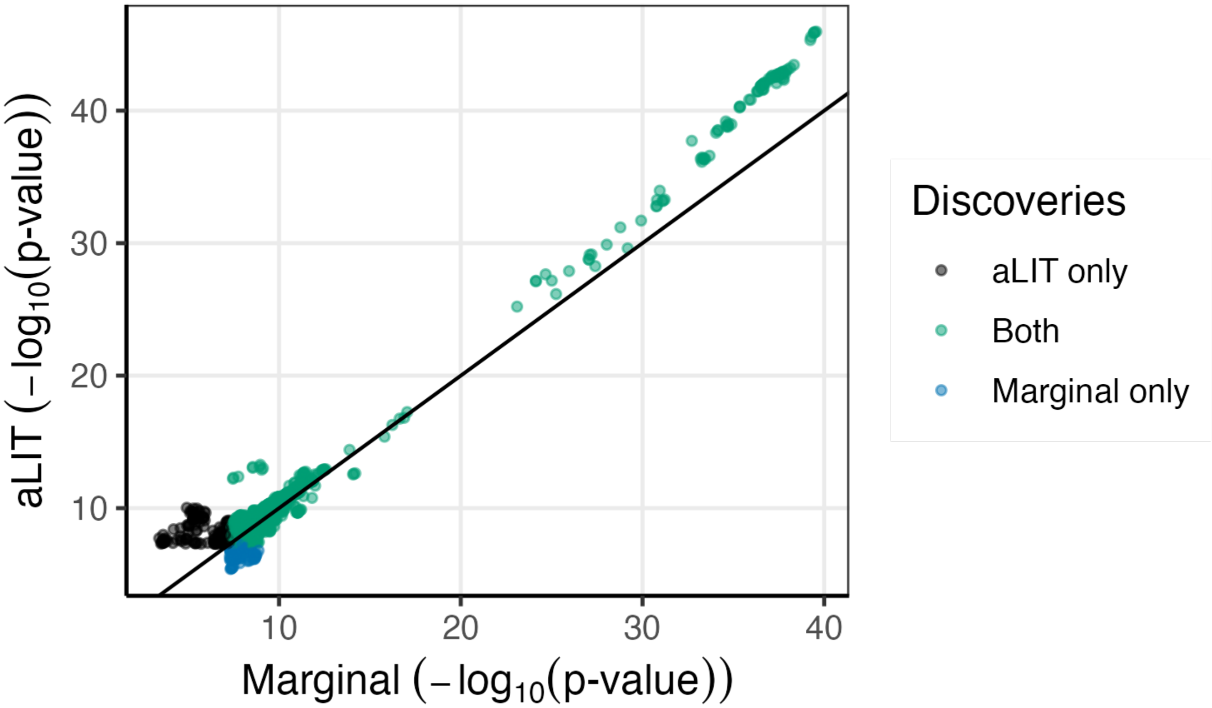

Fig. S14: Comparison of the significance results using the marginal testing procedure and aLIT. The genome-wide significance threshold is 5×10^{-8}.

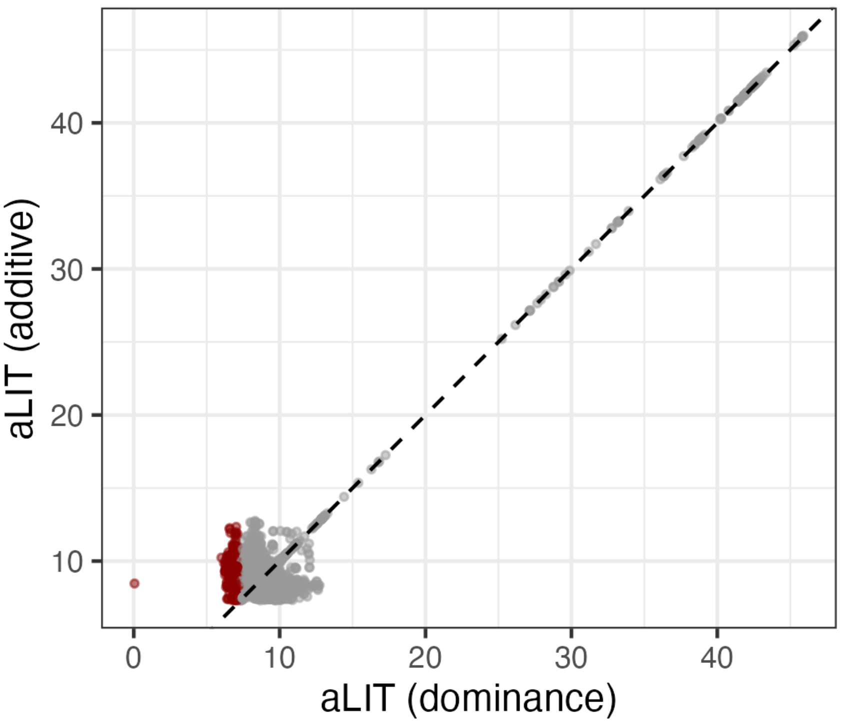

Fig. S15: Comparison of aLIT p-values after adjusting for additive genetic effects (y-axis) and dominance/scaling effects (x-axis). The dark red points are SNPs that are above the genome-wide significance threshold of 5×10^{-8}. The p-values are transformed to be on a logarithmic scale similar to Figure S14.

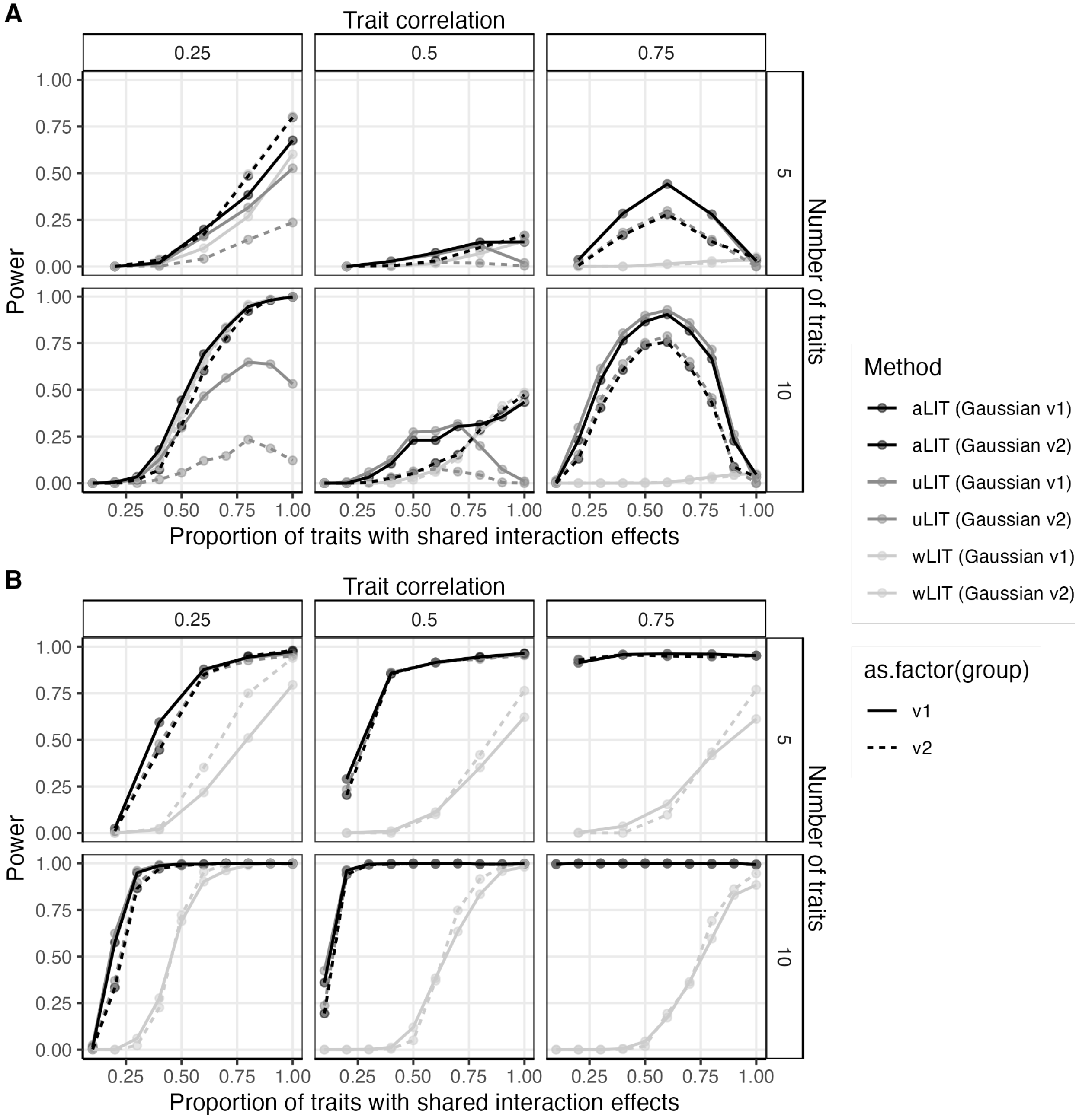

Fig. S16: Implementing an approximate Gaussian kernel to LIT using a similar simulation setting to Figure 2. The 'v1' algorithm assumes a low-rank approximation equal to the number of SQ/CP terms while 'v2' assumes a low-rank approximation equal to three times the number of SQ/CP terms.

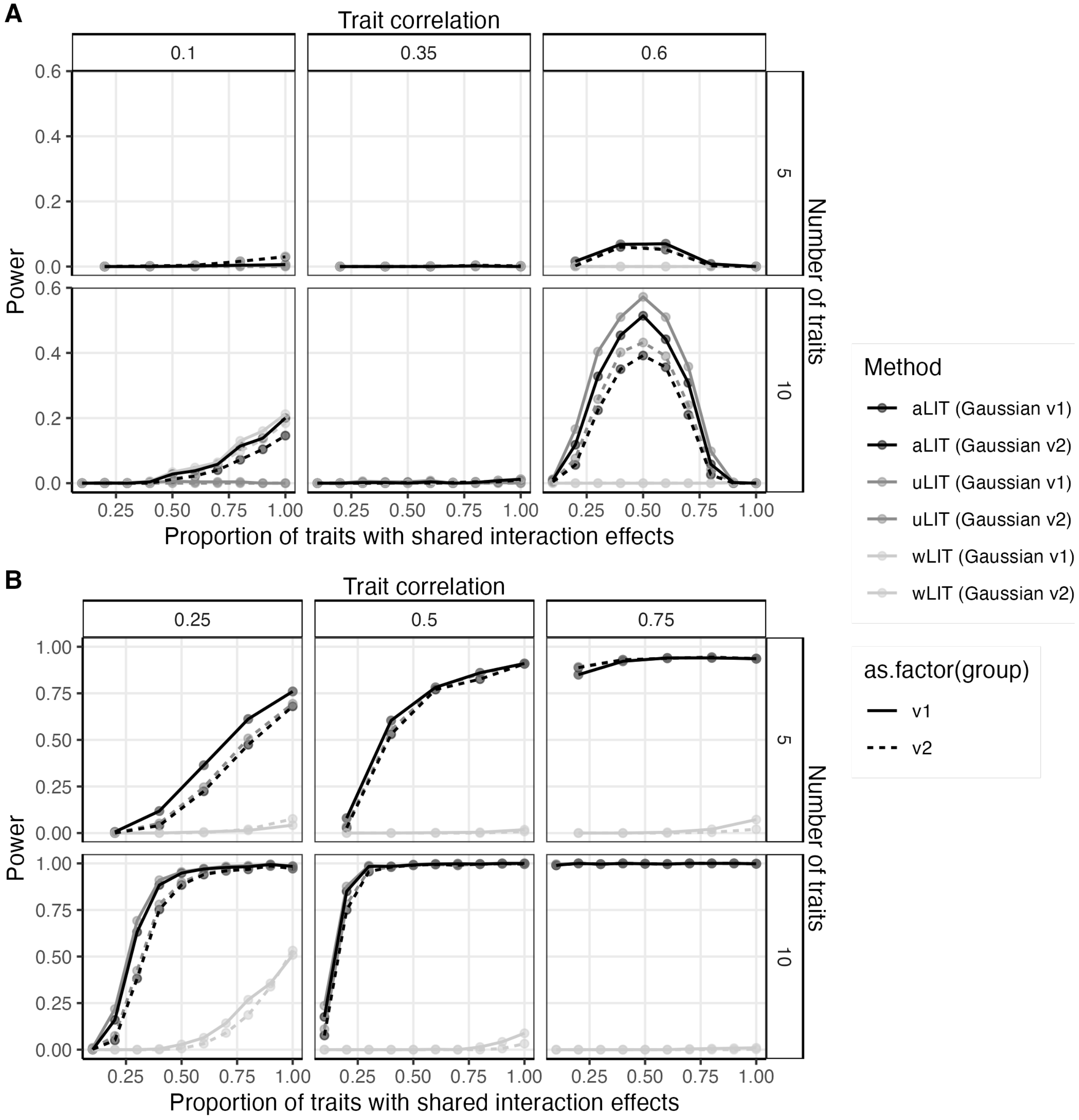

Fig. S17: Implementing an approximate Gaussian kernel to LIT using a similar simulation setting to Figure S7. The ‘v1’ algorithm assumes a low-rank approximation equal to the number of SQ/CP terms while ‘v2’ assumes a low-rank approximation equal to three times the number of SQ/CP terms.

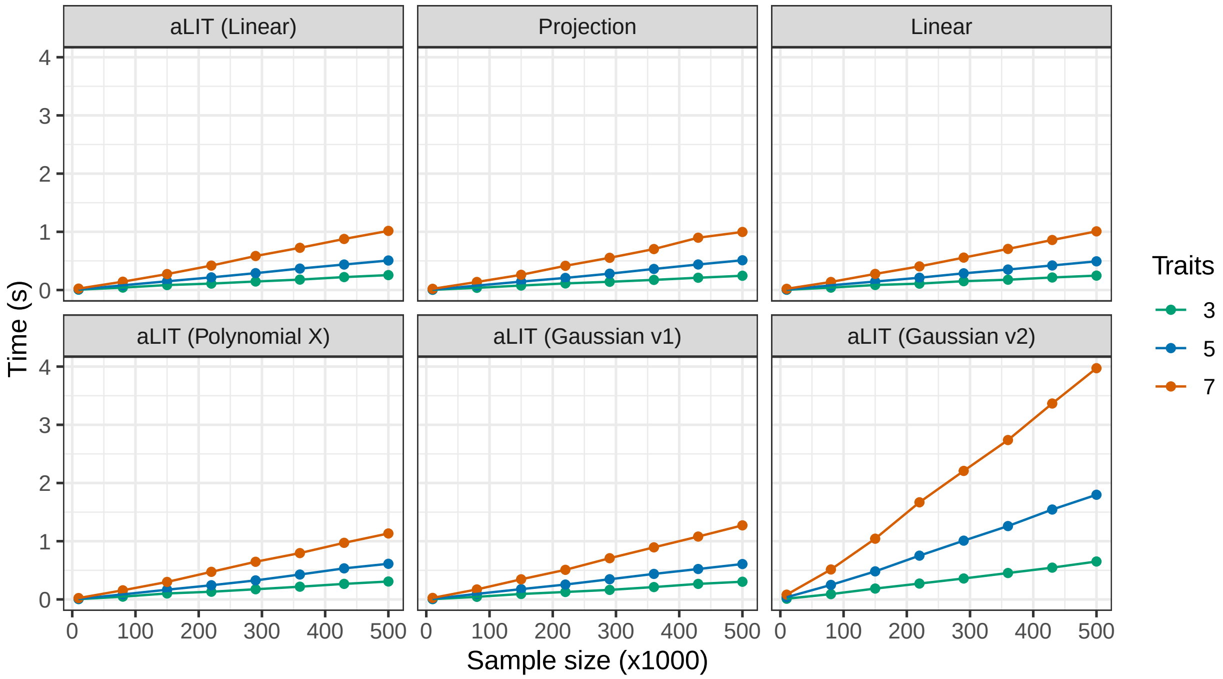

Fig. S18: The average computational time to run aLIT on a SNP as a function of sample size, number of traits, and kernel function. The ‘Projection’ and ‘Linear’ plots show the computational time with the projection and linear kernels, respectively. We also implemented a polynomial kernel for the SNP and an approximate Gaussian kernel for the SQ/CP terms where ‘v1’ and ‘v2’ use a low-rank approximation equal to the number of SQ/CP terms (‘v1’) and three times this values (‘v2’). Data were simulated the same way in the simulation study and each point is the average time across 500 replicates. Note that a single core of a 3.2-GHz Intel Xeon W-3245 processor is used. LIT can distribute across multiple cores to substantially reduce the computational time, and that the relative computation will vary with the computing hardware used.

3 Supplementary tables

Chr.	Gene	Lead SNP	MAF	p-value (aLIT)	p-value (SQ/CP)
16	*FTO*	rs11642015	0.402	1.08×10^{-46}	2.73×10^{-40}
1	*LYPLAL1*	rs2820444	0.299	1.17×10^{-13}	2.64×10^{-13}
6	*BTN2A1*	rs13220495	0.111	2.65×10^{-13}	7.25×10^{-13}
18	*MC4R*	rs6567160	0.234	5.12×10^{-12}	6.58×10^{-12}
6	*SNRPC*	rs4472337	0.155	1.07×10^{-9}	1.99×10^{-10}
2	*COBLL1*	rs10195252	0.407	9.66×10^{-14}	7.64×10^{-10}
7	*KLF14*	rs35363532	0.494	5.75×10^{-10}	8.45×10^{-10}
3	*TIPARP*	rs17451107	0.388	1.57×10^{-7}	1.33×10^{-9}
4	*RP11-362I1.1*	4:45165650_ATTC_A	0.430	5.54×10^{-7}	1.15×10^{-8}
2	*SH3YL1*	rs62104180	0.051	1.34×10^{-7}	1.20×10^{-8}
16	*STX1B*	rs34845977	0.364	1.24×10^{-7}	1.36×10^{-8}
17	*KANSL1*	rs2732706	0.217	1.24×10^{-7}	1.36×10^{-8}
12	*FAIM2*	rs7132908	0.384	8.85×10^{-9}	4.22×10^{-8}

Table S1: Lead SNPs of significant findings from Marginal (SQ/CP) in the UK Biobank.

Chr.	Gene	Lead SNP	age	alcohol	income	sex	smoking
16	*FTO*	rs11642015	6.47×10^{-3}	6.54×10^{-5}	2.27×10^{-1}	1.9×10^{-2}	1.32×10^{-3}
2	*COBLL1*	rs5835988	1.74×10^{-1}	1.72×10^{-1}	2.33×10^{-2}	1.32×10^{-51}	2.46×10^{-3}
1	*LYPLAL1*	rs2820444	9.00×10^{-1}	4.90×10^{-3}	6.50×10^{-1}	7.22×10^{-23}	8.31×10^{-3}
6	*PRSS16*	rs13212921	8.64×10^{-1}	5.26×10^{-1}	6.06×10^{-1}	6.70×10^{-1}	2.23×10^{-1}
18	*MC4R*	rs35614134	7.21×10^{-2}	8.43×10^{-2}	4.59×10^{-1}	6.18×10^{-5}	8.43×10^{-2}
1	*ATP2B4*	rs2821230	6.54×10^{-1}	1.82×10^{-2}	2.78×10^{-1}	1.48×10^{-5}	8.84×10^{-1}
7	*KLF14*	rs972284	1.20×10^{-1}	3.74×10^{-1}	9.80×10^{-1}	2.39×10^{-14}	1.83×10^{-2}
11	*LIN7C*	rs11030066	2.56×10^{-1}	8.76×10^{-1}	2.59×10^{-1}	8.64×10^{-1}	2.13×10^{-1}
6	*ILRUN*	rs9469860	5.40×10^{-1}	9.49×10^{-2}	8.73×10^{-1}	7.87×10^{-5}	1.61×10^{-1}
12	*FAIM2*	rs7132908	4.74×10^{-2}	4.47×10^{-2}	1.02×10^{-1}	3.99×10^{-1}	9.78×10^{-3}
5	*MAP3K1*	rs157845	2.11×10^{-2}	2.71×10^{-1}	1.73×10^{-1}	6.22×10^{-2}	2.12×10^{-2}

Table S2: Genotype-by-environment interaction results for lifestyle (alcohol and smoking) and socio-demographic (age, sex, and income) environmental factors in the UK Biobank.
